# A high copy suppressor screen identifies factors enhancing the allotopic production of subunit II of cytochrome *c* oxidase

**DOI:** 10.1093/g3journal/jkae295

**Published:** 2024-12-13

**Authors:** Felipe Nieto-Panqueva, Miriam Vázquez-Acevedo, David F Barrera-Gómez, Marina Gavilanes-Ruiz, Patrice P Hamel, Diego González-Halphen

**Affiliations:** Instituto de Fisiología Celular, Universidad Nacional Autónoma de México, 04510 Mexico City, Mexico; Instituto de Fisiología Celular, Universidad Nacional Autónoma de México, 04510 Mexico City, Mexico; Departamento de Bioquímica, Facultad de Química, Universidad Nacional Autónoma de México, 04510 Mexico City, Mexico; Departamento de Bioquímica, Facultad de Química, Universidad Nacional Autónoma de México, 04510 Mexico City, Mexico; Department of Molecular Genetics, The Ohio State University, 43210 Columbus, OH, USA; School of BioScience and Technology, Vellore Institute of Technology, 632014 Vellore, Tamil Nadu, India; Instituto de Fisiología Celular, Universidad Nacional Autónoma de México, 04510 Mexico City, Mexico

**Keywords:** subunit 2 of cytochrome *c* oxidase, allotopic expression, protein import into mitochondria, TIM23 translocator, *TYE7*, *RAS2*, COX12

## Abstract

Allotopic expression refers to the artificial relocation of an organellar gene to the nucleus. Subunit 2 (Cox2) of cytochrome *c* oxidase, a subunit with 2 transmembrane domains (TMS1 and TMS2) residing in the inner mitochondrial membrane with a N_out_-C_out_ topology, is typically encoded in the mitochondrial *cox2* gene. In the yeast *Saccharomyces cerevisiae*, the *cox2* gene can be allotopically expressed in the nucleus, yielding a functional protein that restores respiratory growth to a Δ*cox2* null mutant. In addition to a mitochondrial targeting sequence followed by its natural 15-residue leader peptide, the cytosol synthesized Cox2 precursor must carry one or several amino acid substitutions that decrease the mean hydrophobicity of TMS1 and facilitate its import into the matrix by the TIM23 translocase. Here, using a yeast strain that contains a *COX2^W56R^* gene construct inserted in a nuclear chromosome, we searched for genes whose overexpression could facilitate import into mitochondria of the Cox2^W56R^ precursor and increase respiratory growth of the corresponding mutant strain. A *COX2^W56R^* expressing strain was transformed with a multicopy plasmid genomic library, and transformants exhibiting enhanced respiratory growth on nonfermentable carbon sources were selected. We identified 3 genes whose overexpression facilitates the internalization of the Cox2^W56R^ subunit into mitochondria, namely: *TYE7*, *RAS2*, and *COX12*. *TYE7* encodes a transcriptional factor, *RAS2*, a GTP-binding protein, and *COX12*, a non-core subunit of cytochrome *c* oxidase. We discuss potential mechanisms by which the *TYE7*, *RAS2*, and *COX12* gene products could facilitate the import and assembly of the Cox2^W56R^ subunit produced allotopically.

## Introduction

Cytochrome *c* oxidase (C*c*O), or complex IV of the mitochondrial respiratory chain, catalyzes electron transfer from cytochrome *c* to molecular oxygen coupled to proton pumping (Rich 2017). In yeast, C*c*O is formed by polypeptides encoded in 2 different genomes: 3 in the mitochondrial DNA and 8 in the nucleus (Poyton *et al*. 1995). The core of the complex is formed by 3 mitochondria-encoded subunits, Cox1, Cox2, and Cox3, which are normally synthesized by mitochondrial ribosomes and co-translationally inserted into the inner mitochondrial membrane (IMM; Dennerlein *et al*. 2023). The 8 nucleus-encoded subunits, also referred to as noncore subunits, are synthesized in the cytosol as precursors that usually carry a mitochondrial targeting sequence (MTS), imported into mitochondria, proteolytically matured, and assembled into C*c*O (Poyton and Groot 1975; Fontanesi *et al*. 2008). Subunit Cox2 is a membrane-embedded protein with 2 transmembrane stretches (TMS1 and TMS2) and a C-terminal, hydrophilic domain facing the mitochondrial intermembrane space (IMS) that binds a catalytic binuclear copper center. Usually, Cox2 is synthesized by mitochondrial ribosomes as a precursor containing a leader peptide of 15 residues (Torello *et al*. 1997). The TMS1 of the protein is inserted co-translationally into the IMM by the combined action of the mitoribosomes, the IMM-located Oxa1 insertase, and the assembly factors Mba1 and Cox20 (Hell *et al*. 1997, 2000; Herrmann and Bonnefoy 2004; Fiumera *et al*. 2007). When the N-terminus of Cox2 reaches the mitochondrial IMS, the leader peptide is removed by the Imp1 protease (Sevarino and Poyton 1980; Torello *et al*. 1997). By contrast, TMS2 and the large, hydrophilic domain of the protein are translocated across the IMM by Cox18, Pnt1, and Mss2, a process by which the Cox2 subunit exposes the C-terminus to the IMS, thus reaching its characteristic N_out_-C_out_ topology (Saracco and Fox 2002).

Previously, the mitochondrial *cox2* gene was artificially relocated to the nucleus in yeast (Supekova *et al*. 2010). The expression of a *COX2* gene construct from the nucleus complemented a *Δcox2*-null yeast strain, restoring growth in nonfermentable carbon sources. Thus, the cytosol synthesized Cox2 subunit (Cox2^ALLOTOPIC^) was successfully directed to mitochondria and functionally assembled into C*c*O. To be internalized into mitochondria, the allotopically produced Cox2 precursor must exhibit some characteristics: a MTS followed by the natural leader peptide of the yeast mitochondrial Cox2 precursor, and the presence of amino acid substitutions in TMS1, which can be either W56R, W56K, W56Q, or the double V49Q/L51G mutation (Rubalcava-Gracia *et al*. 2018). As expected, the allotopically produced Cox2 protein follows a different biogenesis pathway than the mitochondria synthesized Cox2 subunit, entering mitochondria through the TOM translocator and subsequently sorted by the TIM23 translocator. The amino acid substitutions diminish the mean hydrophobicity of TMS1, promoting its full translocation into the mitochondrial matrix by TIM23. Once the MTS reaches the matrix, it is selectively removed by the mitochondrial processing protease. Then, the Oxa1 insertase recognizes the leader peptide and inserts the TMS1 of the protein into the IMM. When the N-terminus of Cox2 gets exposed to the IMS, Cox20 stabilizes TMS1, and the IMS-localized Imp1 protease removes the leader peptide. Paralleling this process, the highly hydrophobic TMS2 domain of Cox2^ALLOTOPIC^ is retained by the TIM23 complex and released laterally into the IMM, along with the hydrophilic C-terminal domain of the protein, which becomes exposed to the IMS. Thus, the allotopically produced Cox2 subunit reaches the same functional N_out_-C_out_ topology (Nieto-Panqueva *et al*. 2023) attained by its Cox2 counterpart synthesized on mitoribosomes (Rubalcava-Gracia *et al*. 2018). In contrast to the Cox2 protein synthesized in mitochondria, the biogenesis of Cox2^ALLOTOPIC^ does not require the participation of Cox18, which controls the translocation of the TMS2 (Elliott *et al*., 2012). Once the allotopically produced Cox2 protein is inserted in the IMM, it must probably follow the same maturation steps than the Cox2 protein synthesized inside mitochondria: acquisition of a binuclear CuA center (Rigby *et al*. 2008; Pacheu-Grau *et al*. 2015, 2020; Ghosh *et al*. 2016) and its functional assembly into the C*c*O complex (Franco *et al*. 2020).

Although allotopic production of a yeast Cox2 subunit supports respiratory growth, strains expressing *COX2^W56R^* exhibit lower steady-state levels of C*c*O and diminished rates of oxygen uptake when compared with wild-type yeast (Cruz-Torres *et al*. 2012). This phenotype was not due to the presence of the W56R substitution, because when the variant Cox2^W56R^ protein was synthesized inside mitochondria, the resulting activity and levels of C*c*O were found to be equivalent to those of a wild-type strain (Rubalcava-Gracia *et al*. 2018). Thus, the inability of allotopically produced Cox2^W56R^ protein to restore full C*c*O function was attributed to a limiting step during the translocation and sorting of the Cox2^W56R^ precursor by TIM23 (Rubalcava-Gracia *et al*. 2019).

To uncover the factors limiting the ability of cytosol-produced Cox2^W56R^ to fully complement a cox2-null mutant, we undertook 2 approaches. In 1 “educated guess” approach, we tested if overexpression of candidate genes probably involved in the import or assembly of Cox2^W56R^ could improve respiratory growth and found that Mgr2, a regulator of the TIM23 translocon, is instrumental in modulating the import and sorting of Cox2^W56R^ (Nieto-Panqueva *et al*. 2024). Secondly, an unbiased approach was pursued here: using a multicopy genomic library, we searched for multicopy suppressors that could enhance the respiratory growth of the yeast strain expressing the *COX2^W56R^* gene construct from the nucleus (***n**COX2^W56R^*). Overexpression of 3 genes, *COX12*, *RAS2*, and *TYE7* were found to facilitate growth on nonfermentable carbon sources and enhanced levels of mature Cox2^W56R^ subunit in mitochondria. We propose possible mechanisms by which the independent overexpression of these 3 genes promote the internalization into mitochondria of the allotopically produced Cox2^W56R^.

## Materials and methods

### Yeast culture conditions

The yeast strains were cultivated in liquid or solid media containing 2% agar, maintained at 30°C, unless otherwise specified. Media compositions used throughout the study were the following: YPDA (1% yeast extract, 2% bactopeptone, and 2% dextrose) or YPGal (1% yeast extract, 2% bactopeptone, and 2% galactose) as fermentable media; YPEG (1% yeast extract, 2% bacto peptone, 3% ethanol, and 3% v/v glycerol); YPE (1% yeast extract, 2% bacto peptone, 2% ethanol), and YPLAC [3% yeast extract, 1% KH_2_PO_4_, 1% NH_4_Cl, 0.5% CaCl_2_·2H_2_O, 0.5% NaCl, 0.6% MgSO_4_·H2O, 3% FeCl_3_, 2% (v/v) lactate, pH 5.5], as nonfermentable media. Minimal media SD or SGal (0.17% yeast nitrogen base [lacking amino acids and (NH_4_)_2_SO_4_], 0.5% (NH_4_)_2_SO_4_, 2% glucose, or 2% galactose, accordingly, supplemented with specific amino acids and nucleotides), and hygromycin B (300 μg/ml) were used for selection purposes. To monitor growth, optical density at 600 nm (O.D.600) was measured using a Bioscreen C spectrophotometer (Growth Curves, USA).

### Yeast strains and gene constructs

The yeast strains used in this study are derived from the parental strain D273-10B (Fred Sherman lab, ATCC 24657TM). As a wild-type strain, we used NB40-36A 36A (*MATα*; *lys2*; *arg8::his; leu2-3*, *11; ura3-52*; [*rho^+^*]; Pérez-Martínez *et al*. 2003) and as a *cox2*-null strain we used EHW154 (*MATa*; *arg8::hisG*; *ura3-52*; *leu 2-3, 112*; *his3-ΔHindIII*, [*rho^+^*] *cox2(1,89-91)::ARG8m*; Williams and Fox 2003). For *COX2^W56R^* allotopic expression in the Δcox2 mutant, we utilized 2 approaches. Firstly, we introduced the hygromycin B selectable plasmid pRS306H to target insertion via homologous recombination at the URA3 locus (designated as Δ*cox2* + ***n***Cox2^W56R^), and the episomal 2μ URA3 multicopy plasmid pFL61 (termed Δ*cox2* + ***e***Cox2^W56R^; Rubalcava-Gracia *et al*. 2019). The strains used in this work are listed in Supplementary Table 1.

Yeast strains overexpressing *TYE7*, *RAS2*, and *COX12* (designated as *TYE7*↑, *RAS2*↑, and *COX12*↑) were produced by introducing the high copy episomal vector pMK2 with each of the 3 genes cloned. The wild-type *TYE7*, *RAS2*, and *COX12* ORFs were cloned at the *Not*I plasmid multicloning site in pMK2 between the phosphoglycerate kinase (*PGK*) promoter and terminator. The ORFs were PCR-amplified from the chromosomal fragments found in the individual multicopy plasmids recovered from the screen as templates. The following oligonucleotides containing the first 20 bp of each gene fused to a NotI sequence site were used:

TYE7OE: 5´-GCGCGGCCGCATGAACTCTATTTTAGACA-3´ and 5´-GCGCGGCCGCTTATTTTTGGTCTTGTT-3´;
RAS2OE: 5´-GCGCGGCCGCATGCCTTTGAACAAGTC-3´ and 5´-GCGCGGCCGCTTAACTTATAATACAACAGCCACC-3´;
COX12OE: 5´-GCGCGGCCGCATGGCTGATCAAGAAAA-3´ and 5´-GCGCGGCCGCTTAGTCTGAGTTGATATCAC-3´.

The pMK2 plasmid, a derivative of pFL61, features LEU2 as a selectable marker instead of URA3 (Minet *et al*. 1992). The plasmid was constructed by self-ligation of *Bg*lII-cut pFL61, which resulted in the deletion of the *URA3* marker. A 2.8 kb *Bgl*II fragment containing the *LEU2* marker was excised from pFL36 (Bonneaud *et al*. 1991) cloned into the *Bgl*II site of the pFL61-derived plasmid with no *URA3* marker (M. Karamoko, personal communication).

Yeast transformations were conducted employing the 1-step yeast transformation protocol described by Chen *et al*. (1992). Additionally, we utilized the lithium acetate/single-stranded carrier DNA/PEG method of transformation, following the approach detailed by Gietz and Schiestl (2007).

Plasmid manipulation and cloning procedures were used with *Escherichia coli* DH5α (F− endA1 glnV44 thi-1 recA1 relA1 gyrA96 deoR nupG purB20 ϕ80dlacZΔM15 Δ[lacZYA-argF]U169, hsdR17[rK−mK+], λ−) and MR32, a recA− derivative of MC1061 [str. K-12 F− λ− Δ(ara-leu)7697 [araD139]B/r Δ(codB-lacI)3 galK16 galE15 e14− mcrA0 relA1 rpsL150(StrR) spoT1 mcrB1 hsdR2(r−m+)] according to published protocols (Sambrook 2001).

### Multicopy genomic library

The Δ*cox2* + ***n***Cox2^W56R^ was transformed with a yeast genomic library constructed in the pFL44L vector (Bach *et al*. 1979; Oulmouden and Karst 1990). Out of 307,000 primary transformants, 55 colonies displayed enhanced respiratory growth and after re-testing 10 colonies (no. 3 to no. 12, now named MS01 to MS10) exhibited a significant improvement in their ability to grow on a respiratory substrate (glycerol pH: 7.0). For co-segregation experiments, each transformant was grown into liquid YPDA and subcloned to single colony on solid YPDA medium, which was replicated in minimal medium lacking uracil and YPEG medium. The plasmids were recovered from the strains by the glass bead method adapted from Robzyk and Kassir (1992) in *E. coli* MR32. At least 2 independent bacterial clones resulting from transforming the MR32 strain with the extracted DNA from the yeast transformants were selected and the plasmids profiled by restriction digest. Digestion profiles were determined using *Sal*I, *Sph*I, and *Bgl*II, which are present in pFL44L. One profile (Profile 1) defines plasmids from suppressors MS01, MS02, MS03, MS08, MS04, and MS05, a second profile (Profile 2) is found in suppressors MS06 and MS07, and plasmid extracted from MS09 defines a third (Profile 3) distinct profile. Despite numerous attempts, the plasmid contained in the MS10 transformant could never be recovered. The borders of the genomic inserts contained in the 9 pFL44L-based plasmids were sequenced using M13-40 forward and M13-48 reverse primers.

### UV mutagenesis

One UV-induced respiratory-competent revertant was selected from the EHW154 strain. The UV-induced suppressor was isolated from cells grown to stationary phase, which were plated on YPDA medium and stored at 4°C overnight before mutagenesis. UV mutagenesis was performed using a UV source (254 nm) placed at 12 cm from yeast cells. Plates were then irradiated for 10 s, incubated for 3 days in the dark at 28°C, and replica-plated on YPEG. One single revertant was recovered from 2 × 10^9^ cells at an irradiation time causing 30% lethality. The *COX2* ORF in the nucleus was amplified in the suppressed strain using primers PGK-F (5′- CAGATCATCAAGGAAGTAATTATC-3′) and PGK-R (5′- CTATTATTTTAGCGTAAAGGATG-3′) and sequenced to identify the mutation leading to the W56R substitution.

### Growth assessment by 10-fold serial dilutions

Yeast cells were grown overnight in 3 mL of liquid YPDA at 30°C with constant agitation. The following day, the cultures were diluted and grown to an exponential phase (O.D._600_ 1.0). Cells were then centrifuged at 8,600*×g* and washed once with 1 mL of sterile water. This suspension was diluted stepwise 5 times in a 1:10 ratio for each strain and dispensed onto 96-well plates. Using a multichannel pipette, small droplets of roughly 3.5 μL were placed on solid fermentable (YPDA) and non-fermentable (YPEG, YPE, YPLAC) media. The plates were then incubated at 30°C for 4–7 days, as specified.

### RNA extraction and quantitative PCR analysis

Yeast strains were grown in YPGal, and RNA was extracted using TRIzol reagent (Invitrogen, Carlsbad, CA, USA) according to the manufacturer's instructions. The quantity and purity of total RNA were assessed using a NANODROP ND-1000 spectrophotometer (Thermo Scientific Inc., Wilmington, DE, USA). RNA samples with 260/280 nm absorbance ratios close to 2.0 were selected for further analysis (Koetsier and Cantor 2019), and RNA integrity was confirmed by electrophoresis on a 2.0% agarose gel. To eliminate any remaining genomic DNA, RQ1 RNase-Free DNase was added. First-strand cDNA synthesis was performed using 1 µg of RNA, oligo-dT primers, and the ImProm-IITM Reverse Transcription System (Promega, Madison, WI, USA). The resulting cDNA was stored at −20°C until use, then diluted 10-fold, and used as template for quantitative real-time PCR. Amplification was performed using SYBR Green Master Mix (Applied Biosystems) and gene-specific oligonucleotides (Supplementary Table 2) on a micPCR v2.10.0 (Bio Molecular Systems). The genes *ZWF1* (encoding glucose-6-phosphate dehydrogenase), *TDH3* (encoding glyceraldehyde-3-phosphate dehydrogenase isozyme 3), and *CDC19* (encoding pyruvate kinase) were used as housekeeping standards (Cankorur-Cetinkaya *et al*. 2012). The transcript ratios were calculated using the Pfaffl equation (Pfaffl 2001), as modified by Hellemans *et al*. (2007):


$$Ratio\;transcripts=E_{target}^{\Delta CP_{target(control-sample)}\;}/\sqrt[{f}]{\prod_{0}^{f}\;E_{ref_{0}}^{\Delta CP_{ref_{0}}(control-sample)}}\;$$

where E_*target*_ is the amplification efficiency of the target gene, ΔCP_*target*_ is the Ct difference between control and samples where genes were overexpressed, E*ref*_0_ is the amplification efficiency of the housekeeping gene, and Δ*CPref*_0_ is the Ct difference between control and overexpressing samples. Statistical analysis was performed using the MetaboAnalyst 6.0 software. Mean values, standard error, and 1-way analysis of variance (ANOVA) were calculated to identify significant differences, with a significance threshold set at α = 0.05. Post hoc comparisons were conducted using Tukey's test.

### Protein extraction and immunodetection

Alkaline treatment was employed for protein extraction from whole yeast cells cultivated in 20 mL of liquid SGal media to the stationary phase (OD600 2.5). The cells were then collected by centrifugation at 2,800*×g* for 3 minutes in a microcentrifuge. Cells were then washed using a 500 μl solution containing 1 mM phenylmethylsufonyl fluoride (PMSF) and 50 mM N-α-tosyl-L-lysyl-chloromethyl ketone. The pellet was boiled in SDS-PAGE sample buffer (100 mM Tris–HCl pH 6.8, 10% SDS, 30% glycerol, 4% β-mercaptoethanol, 0.3% bromophenol blue) following the protocol by Kushnirov (2000). Gel electrophoresis was performed using an SDS-tricine-PAGE system as described by Schägger (1994). After electrophoresis, the gels were transferred to nitrocellulose membranes, which were then blocked with 1% bovine skin type B gelatin (Invitrogen), followed by washing and subsequent immuno-decoration. The monoclonal αCox2 antibody (Abcam: 4B12A5) was used at a 1:9,000 dilution, as detailed in Ghosh *et al.* (2016). As a loading control, a polyclonal αZwf1 antibody (Sigma-Aldrich: A9521) was employed at a 1:10,000 dilution (Fujiwara *et al*. 2016). Secondary antibody labeling involved a 4-hour incubation with alkaline phosphatase-conjugated goat anti-rabbit and goat anti-mouse antibodies. The appearance of dark purple precipitates in band form was observed after adding nitro-blue tetrazolium chloride and 5-bromo-4-chloro-3′-indolyl phosphate *p*-toluidine salt. An HP Scanjet G4050 image scanner was utilized to capture the colored bands formed by the precipitate on the membranes. The intensity of the colored bands was estimated using the GelAnalyzer 23.1 freeware developed by Istvan Lazar Jr., PhD and Istvan Lazar Sr., PhD, CSc. (www.gelanalyzer.com). For bar plotting and statistical analysis GraphPad Prism version 10.1.1(27) for macOS (GraphPad Software, Boston, MA, USA, www.graphpad.com) was used. For each subset of samples, a 2-way ANOVA followed by Tukey's multiple comparisons test was carried out.

### In silico analyses

The genes networks up-regulated by the transcription factor Tye7 were searched in YEASTRACT (Yeast Search for Transcriptional Regulators And Consensus Tracking), a curated repository of regulatory associations between transcription factors and target genes in *Saccharomyces cerevisiae* (http://yeastract.com/index.php; Teixeira *et al*. 2023). Physical and genetic interactions of Tye7 were explored in the yeast interactome (Cherry *et al*. 1997) at the Saccharomyces Genome Database (https://www.yeastgenome.org/).

## Results

**Fig. 1.**
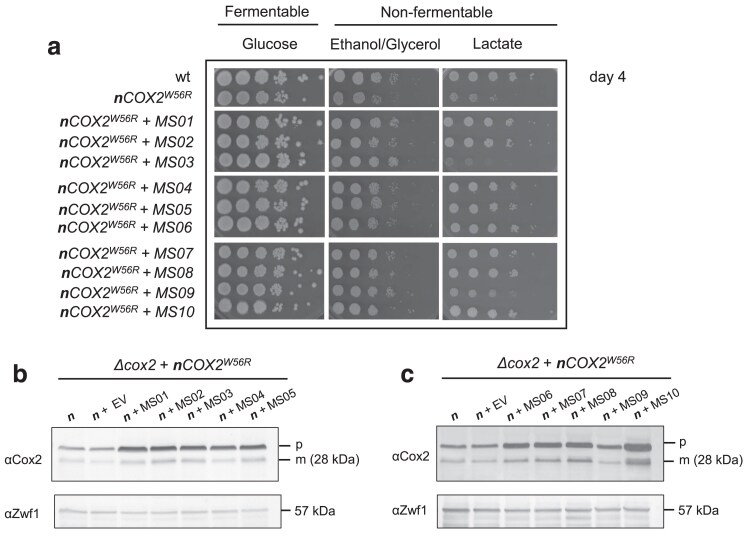
Ten transformants (MS01 to MS10) exhibited a significant improvement in their respiratory growth and increased levels of mature Cox2 subunit. a) Serial dilutions at 30°C showing the fermentative (glucose) and respiratory (ethanol/glycerol and lactate) growth phenotype of a wild-type strain (wt), the *Δcox2* + *nCOX2^W56R^* strain (*nCOX2^W56R^*), and the 10 strains (MS01 to MS10) obtained after transformation with the multicopy genomic library. Photographs were taken on the fourth day of growth. b and c) Antibodies against Cox2 and Zwf1 were used to immunodetect the corresponding proteins in total cellular extracts of the indicated yeast strains: the *Δcox2* + *nCOX2^W56R^* strain (*n*), the control strain transformed with an empty vector (*n* + EV), and the 10 transformants (MS01 to MS10). The precursor (p) and mature (m) forms of Cox2^W56R^ are indicated. The anti-Zw1 antibody immunoreacts against a glucose-6-phosphate dehydrogenase (57 kDa band) that was used as a loading control.

To uncover the factors limiting the ability of cytosol-produced *Cox2^W56R^* to fully complement a *cox2*-null mutant, we searched for high-copy suppressors that could improve the respiratory growth of a strain expressing an allotopic *COX2^W56R^* construct from the nucleus (*n*Cox2^W56R^). For this purpose, the *nCOX2^W56R^* strain was transformed with a yeast genomic library containing chromosomal fragments inserted into the multicopy pFL44L vector. Out of an estimated number of 307,000 primary transformants, 55 colonies displayed enhanced respiratory growth, and after retesting, 10 colonies (named as Multicopy Suppressors MS01 to MS10) were selected, since they exhibited a significant improvement in their ability to grow on nonfermentable carbon sources when compared with the original ***n**COX2^W56R^* strain (Fig. 1a). Some transformants exhibited an improved respiratory growth on ethanol/glycerol but not on lactate. The reason for this carbon source dependent difference in respiratory growth is not obvious. Immunoblotting analysis revealed that overexpression of the chromosomal fragments in the 10 transformants increased the levels of both the precursor and mature forms of *n*Cox2^W56R^ (Figs. 1b and 1c) when compared with a strain carrying the empty vector (*n* + EV).

For each multicopy suppressor transformant (MS01 to MS10), a colony that had lost the plasmid (-ura) was retested for the presence of the *arg8m* and *hph* markers, as well as enhanced respiratory growth. The 10 colonies were uracil auxotrophs, and concomitantly, had lost the enhanced respiratory growth phenotype (data not shown). By contrast, all plasmid containing colonies displayed enhanced respiratory growth compared with the recipient strain (***n**COX2^W56R^*), suggesting that this trait was plasmid-borne for all the isolated multicopy suppressors (data not shown). Altogether, these co-segregation experiments showed that the enhanced respiratory ability was indeed conferred by a multicopy plasmid derived from the pFL44L-based genomic library.

**Fig. 2.**
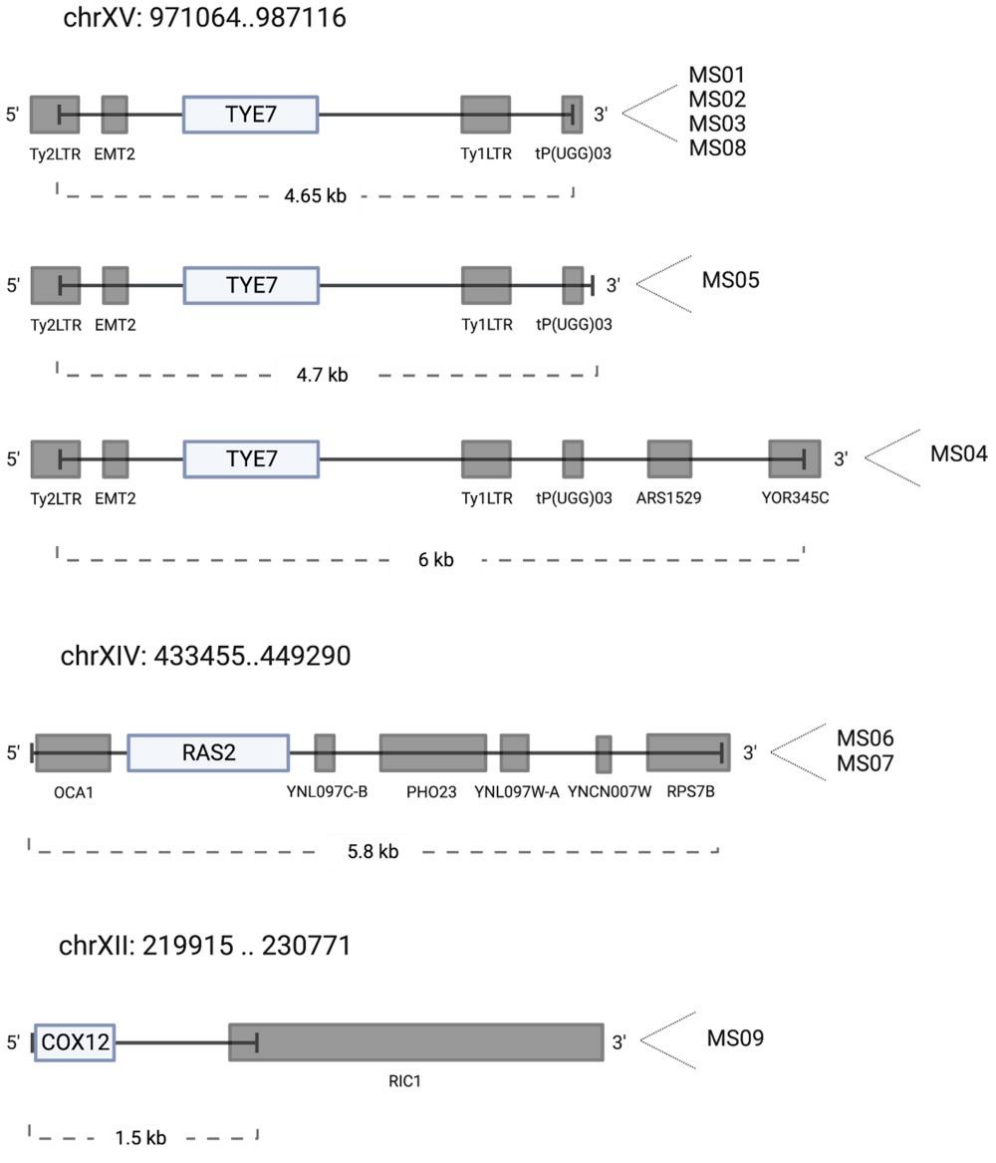
Three distinct chromosomal fragments are present in the multicopy plasmids in transformants M01 to M09. The scheme depicts the genomic fragments from chromosomes XV, XIV, and XII (with corresponding coordinates) cloned in pFL44L-based plasmids carried by the selected transformants and the ORFs present in each one of them (gray and light gray boxes). The 3 candidate ORFs selected for further analysis are marked as *TYE7*, *RAS2*, and *COX12*.

Plasmid DNA was recovered from 9 of the 10 yeast multicopy suppressors (MS01 to MS09), and 3 different restriction profiles were identified. The first profile was found in suppressors MS01 to MS05 and MS08, the second profile was found in suppressors MS06 and MS07, and the third one in MS09, which appeared to correspond to a re-arranged plasmid. The borders of the inserts from 5 of the multicopy plasmids were sequenced and 4 chromosomal fragments were identified, along with the ORFs present in each one of them (Fig. 2). This analysis led to the identification of 3 candidate ORFs conferring the suppression in the chromosomal fragments: *TYE7* (YOR344C), present in the plasmid contained in transformants MS01 to MS05 and MS08; *RAS2* (YNL098C), present in transformants MS06 and MS07; and *COX12* (YLR038C), present in transformant MS09.

**Fig. 3.**
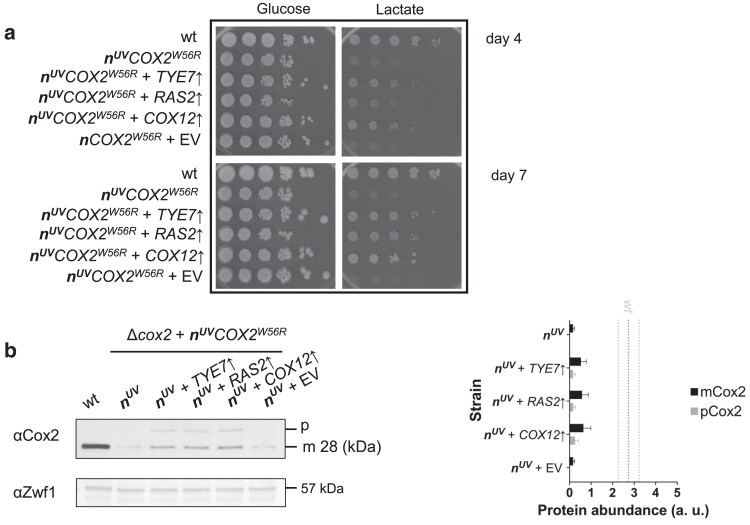
Overexpression of the *TYE7*, *RAS2*, and *COX12* genes affects the growth of a strain expressing the *COX2^W56R^* gene from the nucleus. a) Serial dilution series showing the fermentative (glucose) and respiratory (ethanol/glycerol, ethanol, and lactate) growth phenotypes of a wild-type strain (wt), the *Δcox2* + *n^uv^COX2^W56R^* strain (*n^uv^COX2^W56R^*) obtained after UV mutagenesis, and the 3 strains overexpressing (↑) the indicated gene or carrying the empty vector (EV) in the *n^uv^COX2^W56R^* background at 30°C. Photographs were taken on the fourth day (upper panels) and on the seventh day of growth (lower panels). b) Antibodies against Cox2 and Zwf1 were used to immunodetect the corresponding proteins in total cellular extracts of the indicated yeast strains: wild type (wt); the *Δcox2* + *n^uv^COX2^W56R^* strain (*n*), the 3 strains overexpressing (↑) the indicated gene on a *n^uv^COX2^W56R^* background, and the control strain transformed with an empty vector (*n ^uv^* + EV). The mature proteins Cox2 or Cox2^W56R^ (28 kDa) are indicated. The anti-Zw1 antibody immunoreacts against a 57 kDa band, which is used as a loading control. Quantification of technical replicates of immunoblots are represented by bar plots (mean ± SD, *n* = 3). The observed increased Cox2 levels relative to the empty vector control sample were statistically significant (*P* < 0.001). Black and grey dotted lines indicate the mean and SD abundance of the wild-type protein. *Δcox2* data are not displayed.

To assess if the 3 candidate genes were indeed responsible for causing the observed phenotype, i.e. enhanced respiratory growth, the 3 ORFs were cloned downstream of a strong, constitutive promoter (*PGK*) in a different multicopy plasmid (pMK2). Thus, the original ***n**COX2^W56R^* strain was independently transformed with the 3 candidate genes, each one cloned in the pMK2 vector. The growth of the nontransformed ***n**COX2^W56R^* strain was compared with that of the 3 transformants overexpressing *TYE7*, *RAS2*, and *COX12*. Unexpectedly, none of the overexpressed genes caused a significant growth improvement on respiratory media (Supplementary Fig. 1). We reasoned that the original strain containing the ***n**COX2^W56R^* gene construct integrated in the nucleus might have been silenced or could had evolved over time, reaching maximal growth efficiency, which did not enhance further upon the independent overexpression of the 3 candidate genes. This finding led us to generate a new allotopic strain to explore the effects of overexpressing the 3 candidate genes in a different genetic background. For this purpose, a yeast strain carrying the mitochondrial *cox2* null mutation and a nucleus localized wild-type *COX2* gene (lacking the mutation corresponding to the W56R substitution) was subjected to UV irradiation. One mutant strain that was able to grow on respiratory was recovered, and we hypothesized that the UV induced mutation occurred in the *COX2* gene. Sequence analysis showed that indeed, UV light treatment induced a mutation in the *COX2* gene that gave rise to a W56R substitution in the corresponding protein product. The ***n^uv^**COX2^W56R^* strain was also independently transformed with pMK2 plasmids containing the 3 candidate genes. The growth of the nontransformed ***n^uv^**COX2^W56R^* strain and the 3 transformants overexpressing *TYE7*, *RAS2*, and *COX12*, and the ***n^uv^**COX2^W56R^* strain transformed with an empty pMK2 plasmid were compared. The transformants overexpressing the candidate genes exhibited a significant improvement in their growth in respiratory media (Fig. 3a). In addition, immunoblotting analysis revealed that overexpression of the 3 candidate genes in the ***n^uv^**COX2^W56R^* strain led to increased levels of the mature form of the Cox2^W56R^ subunit (Fig. 3b).

**Fig. 4.**
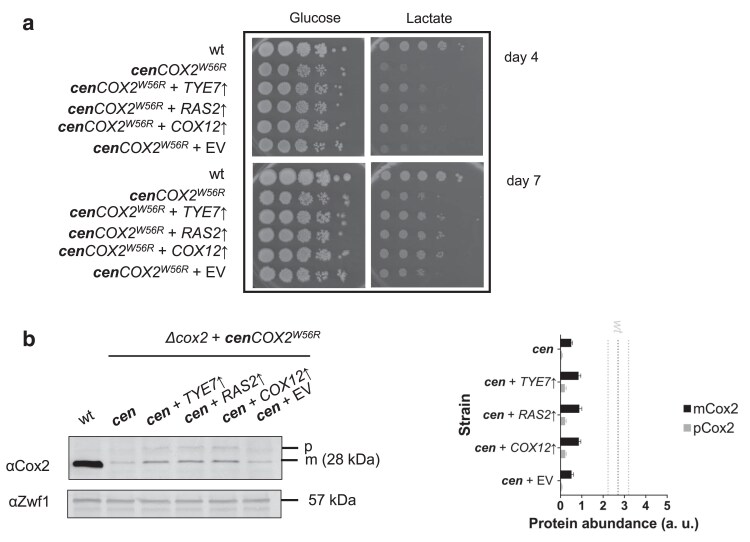
Overexpression of the *TYE7*, *RAS2*, and *COX12* genes enhance the respiratory growth of a strain expressing the *COX2^W56R^* gene from a centromeric plasmid. a) Serial dilution series showing the fermentative (glucose) and respiratory (ethanol/glycerol, ethanol, and lactate) growth phenotypes of a wild-type strain (wt), the *Δcox2* + *cenCOX2^W56R^* strain (*cenCOX2^W56R^*), and the 3 strains overexpressing (↑) the indicated gene or carrying the empty vector (EV) in the *cenCOX2^W56R^* background at 30°C. Photographs were taken on the fourth day (upper panels) and on the seventh day of growth (lower panels). b) Antibodies against Cox2 and Zwf1 were used to immunodetect the corresponding proteins in total cellular extracts of the indicated yeast strains: wild type (wt); the *Δcox2* + *cenCOX2^W56R^* strain (*cen*), the 3 strains overexpressing (↑) the indicated gene on a *cenCOX2^W56R^* background, and the control strain transformed with an empty vector (*cen* + EV). The mature proteins Cox2 or Cox2^W56R^ (28 kDa) are indicated. The anti-Zw1 antibody immunoreacts against a 57 kDa band, which is used as a loading control. Quantification of technical replicates of immunoblots are represented by bar plots (mean ± SD, *n* = 3). The observed increased Cox2 levels relative to the empty vector control sample were statistically significant (*P* < 0.001). Black and grey dotted lines indicate the mean and SD abundance of the wild-type protein. *Δcox2* data is not displayed.

To test whether the high-copy suppressors improved the growth of *COX2^W56R^* strain, we also transformed a strain expressing the *COX2^W56R^* construct from a centromeric plasmid (***cen**COX2^W56R^*), which should generate levels of cytosolic Cox2 precursor protein like those present in an ***n**COX2^W56R^* strain. Thus, the strain ***cen**COX2^W56R^* was independently transformed with the 3 candidate genes, each one cloned in the pMK2 vector. The growth of the nontransformed *COX2^W56R^* strain and the 3 transformants overexpressing *TYE7*, *RAS2*, and *COX12* were compared, as well as a ***cen**COX2^W56R^* transformed with an empty pMK2 plasmid. The transformants overexpressing the candidate genes exhibited a reproducible and significant improvement in their growth on respiratory media (Fig. 4a). In addition, immunoblotting analysis revealed that the 3 high-copy suppressors in the ***cen**COX2^W56R^* strain increased the levels of the mature form of Cox2^W56R^ (Fig. 4b). The independent overexpression of the *TYE7*, *RAS2*, and *COX12* genes was also evaluated at 37°C. Overexpression of *TYE7* and *RAS2* enhanced the growth of the ***cen**COX2*^W56R^ strain at this elevated temperature, whereas *COX12* overexpression had no noticeable effect on growth compared to the strain containing the empty vector (Supplementary Fig. 2).

**Fig. 5.**
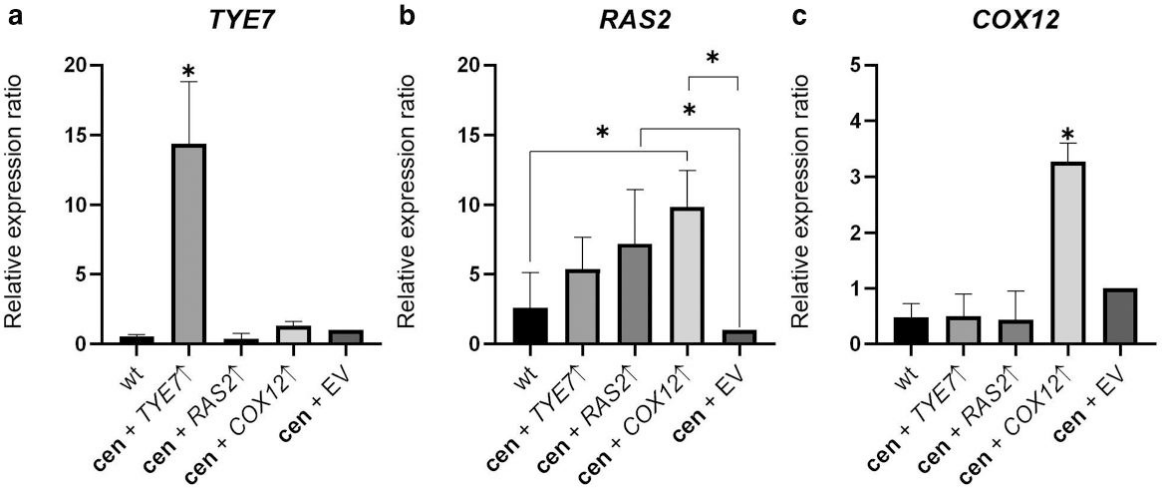
Relative expression levels estimated by qPCR analysis of the *TYE7*, *RAS2*, and *COX12* genes in the control and overexpressing strains. The relative expression of the 3 genes were estimated in the 3 *cenCOX2^W56R^* strains independently overexpressing *TYE7* (a), *RAS2* (b), or *COX12* (c), and compared to those of the WT strain and the *Δcox2* + *cenCOX2^W56R^* carrying the empty vector strain (*cen* + EV). Expression levels were normalized to 3 house keeping genes and are presented as fold changes relative to the control. Error bars correspond to 4 technical duplicates from 2 biological duplicates and asterisks indicate statistical significance (*P* < 0.05).

To determine whether the candidate genes *TYE7*, *RAS2*, and *COX12* were overexpressed in the ***cen**COX2*^W56R^ strain, we compared their relative expression by quantitative PCR (qPCR) in the 3 transformants, using both the empty vector strain and the wild-type yeast as controls. Our findings confirmed that all 3 genes were overexpressed relative to the empty vector control, though to varying extents: *TYE7* was overexpressed 14-fold, *RAS2* 5-fold, and *COX12* 3.5-fold (Fig. 5).

It was previously shown that directing high doses of the Cox2^ALLOTOPIC^ subunit to mitochondria (i.e. using an expression system with a multicopy vector) could be deleterious because the subunit aggregates at the mitochondrial surface (Rubalcava-Gracia *et al*. 2019). Following the same line of reasoning concerning dosage of the cytosolically produced Cox2^W56R^ protein, we also explored the effect of overexpressing the *TYE7*, *RAS2*, and *COX12* genes on an ***e**COX2^W56R^* background, i.e. a strain expressing the allotopic gene from a multicopy plasmid. Only the transformants overexpressing *TYE7* and the *RAS2* genes induced enhanced growth of the strain on lactate (Supplementary Fig. 3a), which correlated with lower levels of the Cox2^W56R^ precursor and higher levels of its mature form (Supplementary Fig. 3b).

To explore whether overexpression of the *TYE7*, *RAS2*, and *COX12* genes could also influence the expression of mitochondrial genes, we compared the relative expression levels of the *cox1* and *cox3* genes by qPCR in the 3 overexpressing strains. All 3 nucleus-located genes failed to increase the levels of the 2 mitochondrial genes encoding C*c*O subunits (data not shown).

## Discussion

The functional transfer of a mitochondrial gene to the nucleus, known as allotopic expression, is considered a potential technique to develop therapies to address human diseases related to mutations in mitochondrial genes. For this process to be successful, the cognate protein must be synthesized in the cytosol, imported into its destined mitochondrial sub compartment, and functionally assembled into its corresponding protein complex. Many reports of allotopic expression of several mitochondrial genes from diverse biological systems have been put forward, but few of these claims have been confirmed biochemically (Nieto-Panqueva *et al*. 2023). One of the exceptions is the allotopically produced yeast Cox2 subunit, whose import pathway into mitochondria has been extensively characterized. In this work, we searched for factors that may further enhance the import and assembly of a Cox2^ALLOTOPIC^ subunit into mitochondria.

Here, we identified 3 genes (*TYE7*, *RAS2*, and *COX12*) whose overexpression promotes higher respiratory growth of a *COX2^W56R^* strain, which correlates with enhanced levels of mature allotopic Cox2^W56R^ protein inside mitochondria. As stated below, we propose possible mechanisms through which the protein products encoded in these overexpressed genes may facilitate the internalization of the allotopic subunit into mitochondria.

### Overexpression of the TYE7 gene facilitates the internalization of the allotopic Cox2^W56R^ subunit into mitochondria

Overexpression of the *TYE7* gene increased the levels of mature Cox2^W56R^ in mitochondria. *TYE7* encodes a 33 kDa member of the basic region/helix-loop-helix/leucine-zipper protein family that exhibits a N-terminal region extremely rich in serine and a C-terminal sequence that has similarity with Myc and Max proteins (Löhning and Ciriacy 1994). The Tye7 protein functions as a transcriptional activator in retrotransposon Ty1-mediated gene expression (Servant *et al*. 2012; Gui *et al*. 2021). Through binding to E-boxes, Tye7 activates several genes encoding glycolytic enzymes like enolase I, 3-phosphoglycerate kinase, pyruvate kinase, glyceraldehyde-3-phosphate dehydrogenase, phosphoglycerate mutase, and triosephosphate isomerase (Nishi *et al*. 1995; Sato *et al*. 1999; Robinson and Lopes 2000). Tye7 is also predicted to activate a series of genes encoding components related to the mitochondrial protein import machinery (Horak *et al*. 2002; Reimand *et al*. 2010; Holland *et al*. 2019) including the Oxa1 insertase, Cox18 involved in the biogenesis of the mitochondrial Cox2 protein; Tom40, a component of the TOM complex; Tim50 and Ssc1, 2 components of the TIM23 complex; as well as several chaperones of the HSP70 family, i.e. Sse2, Ssb1, and Mdj1 (Supplementary Table 3). This suggests that overexpression of *TYE7* may promote Cox2^W56R^ internalization into mitochondria through an indirect mechanism, possibly by activating the expression of several genes whose protein products are involved in mitochondrial protein import.

### Overexpression of the RAS2 gene raises the levels of the allotopic Cox2^W56R^ subunit in mitochondria

Overexpression of the *RAS2* gene was also found to increase the growth of yeast strains producing the allotopic Cox2^W56R^ subunit, whether the gene was inserted in the nucleus, in a centromeric, or in a multicopy plasmid. The Ras superfamily comprises >150 small GTPases that share structural and biochemical properties, mediating multiple metabolic responses and acting as key regulators in different signaling routes (Broach and Deschenes 1990; Goitre *et al*. 2014), including control of mitochondrial biogenesis and function (Hlavatá and Nyström 2003). When yeast grows using glucose as a carbon source, Ras proteins (the 2 isoforms Ras1 and Ras2) accumulate preferably in both the plasma membrane and in the nucleus. Nevertheless, in the absence of glucose, Ras proteins tend to accumulate in mitochondria (Broggi *et al*. 2013). Furthermore, deletion of the *RAS2* gene impedes yeast growth in nonfermentable carbon sources (Tatchell *et al*. 1985). The Ras2 protein activates adenylyl cyclase and other protein kinases that rely on cAMP (Cannon and Tatchell, 1987), like the mitochondria-localized protein kinase A (PKA; Papa *et al*. 1996). Nutrient availability and the signaling route RAS/cAMP play a critical role in the cellular growth of *S. cerevisiae* (Matsumoto *et al*. 1983). Early on, it was observed that in yeast, cAMP reverses the glucose repression on mitochondrial biogenesis (Fang and Butow 1970) and activates mitochondrial genes encoding C*c*O subunits and other OXPHOS components (Chandrasekaran and Jayaraman 1978). Therefore, along with PKA, cAMP seems to govern mitochondrial transcription (Müller and Bandlow 1987) by acting on a *cis*-regulatory element present in mitochondrial DNA (Iqbal *et al*. 1996). In mammals, the cAMP/PKA pathway was shown to regulate the biogenesis, assembly, and catalytic activity of respiratory complex I (Papa *et al*. 2012). In addition, *RAS2* overexpression suppresses a mutation of citrate synthase (*CIT2*), a participant of the glyoxylate cycle (Swiegers *et al*. 2006) and suppresses a mutation in a noncatalytic site of the alpha subunit (atp1-2) of mitochondrial ATP synthase (Mabuchi *et al*. 2000). Also, RAS2^Val19^ cells exhibiting an overactive RAS/PKA signaling abrogate activity of the mitochondrial ATP/ADP carrier (Hlavatá *et al*. 2008). Some of these findings seem to be related to an induced expression of ribosomal protein genes (Neuman-Silberberg, *et al*. 1995) linked to an increased transcription rate of nuclear genes encoding mitochondrial proteins. Overall, the Ras/adenylate cyclase/PKA pathway is involved in the regulation of metabolism, adaptation to glucose, proliferation, cell growth, stress resistance, in mediating the response to the availability of nutrients, and controlling cytosolic cAMP levels and PKA activity (Tisi *et al*. 2014). This signaling route also regulates proteins involved in other mitochondria-related phenomena like apoptosis and mitochondrial fission (Cribbs and Strack 2007; Ould Amer and Hebert-Chatelain, 2018). In yeast mutants exhibiting alterations in this signaling pathway, it was shown that high intracellular levels of cAMP increase the relative abundance of OXPHOS complexes and the mitochondrial respiratory activity (Dejean *et al*. 2002). Thus, a tight link exists between the activity of the Ras/adenylate cyclase/PKA pathway and the levels of OXPHOS complexes (Bouchez and Devin 2019). The increased cell levels of cAMP enhance the stability of the transcriptional co-activator Hap4p, a key regulator of the HAP complex (Bouchez *et al*. 2020). HAP transcriptional complex is an instrumental factor for yeast growth on respiratory substrates, since it controls mitochondrial translation, reprogramming the yeast cell from fermentation to respiration and serving as a coordinator of mitochondrial and nuclear gene expression (Buschlen *et al*. 2003). Overall, an increase in the activity of the cAMP pathway positively regulates mitochondrial biogenesis (Yoboue *et al*. 2012). Given the wide range of effects that Ras proteins regulatory pathways have on several nucleus and mitochondria encoded enzymes, we initially thought that the overexpression of *RAS2* might promote increased transcription of several mitochondrial OXPHOS genes, including *cox1* and *cox3*, with the concomitant increase in the levels of Cox1 and Cox3 modules in the IMM. These modules could readily integrate incoming allotopic Cox2^W56R^ subunits to form fully assembled C*c*O complexes. Nevertheless, qPCR experiments showed no increased levels of expression of the mitochondrial *cox1* and *cox3* genes in the strain overexpressing the *RAS2* gene as compared to the control (data not shown). Further exploration is needed to elucidate the effect of *RAS2* on the import and assembly of the allotopically produced Cox2 subunit.

### Overexpression of the COX12 gene facilitates the internalization of the allotopic Cox2^W56R^ subunit into mitochondria

The *COX12* gene encodes subunit VIb (also known as Cox12), a loosely bound and soluble subunit of C*c*O facing the IMS (LaMarche *et al*. 1992; Ing *et al*. 2022). The Cox12 subunit is required for the formation of active C*c*O and its absence results in the assembly of an optically detectable C*c*O with a markedly diminished activity. Cox12 interacts with both Rcf1 and Rcf2, which are required for respiratory complex biogenesis and act mainly by regulating the assembly and enzyme activity of complex IV within supercomplexes, suggesting a role for this C*c*O subunit in super complex formation (Das *et al*. 2021). Recent genetic studies suggest that Cox12 participates in the insertion of the binuclear copper center in the Cox2 subunit along with the assembly factor Coa6. While the loss of Coa6 or Cox12 can be rescued by copper supplementation, elimination of both Coa6 and Cox12 completely abrogates Cox2 biogenesis, and copper supplementation fails to rescue Cox2 levels (Ghosh *et al*. 2014, 2016). The overproduction of the *COX12* gene partially rescued the *coa6* null mutant. Physical interactions were observed between Coa6, Cox2, Cox12, and the Sco proteins, which are involved in copper delivery to Cox2. It is possible that the overexpression of the *COX12* gene suppresses the Δ*coa6* mutant by stabilizing and enhancing the formation of the binuclear CuA site. Copper insertion into the metal center is probably a limiting step in Cox2 biogenesis, due to the several factors that participate in the formation of its metal center, which must also minimize the production of reactive C*c*O assembly intermediates (Nývltová *et al*. 2022). We, therefore, suggest that high levels of the Cox12 subunit in the IMS could speed-up copper delivery and assembly of the CuA center, facilitating the subsequent incorporation of Cox2^W56R^ into C*c*O, and simultaneously enhancing the internalization rate of its precursor into mitochondria.

In summary, we identified 3 yeast genes that when overexpressed, facilitate the internalization of the allotopically produced Cox2^W56R^ protein into mitochondria. Although the suggested mechanisms by which the Tye7, Ras2, and Cox12 independently exert their effect are different, they all seem to facilitate either the internalization or the assembly line of the allotopic Cox2 subunit.

## Supplementary Material

jkae295_Supplementary_Data

## Data Availability

Strains and plasmids are available upon request. The authors affirm that all data necessary for confirming the conclusions of the article are present within the article, figures, and tables. Supplemental material available at G3 online.
