# Supplementary material for: A high copy suppressor screen identifies factors enhancing the allotopic production of subunit II of cytochrome c oxidase
Source: G3 (Bethesda). 2024 Dec 13;15(3):jkae295. doi: 10.1093/g3journal/jkae295 (PMC11917479; doi:10.1093/g3journal/jkae295)
Supplement: jkae295_Supplementary_Data [file jkae295_supplementary_data.zip › Supplemental_Figure_Legends_G3-2024-405571.docx]

**LEGENDS TO SUPPLEMENTARY FIGURES:**

Figure S1. **Overexpression of the *TYE7*, *RAS2*, and *COX12* genes** **does not improve the growth of a strain expressing the *COX2^W56R^* gene from the nucleus.** Serial dilution series showing the fermentative (glucose) and respiratory (lactate) growth phenotypes of a wild-type strain (wt), the *∆cox2* + ***n****COX2^W56R^* strain (***n****COX2^W56R^*), and the three strains overexpressing (↑) the indicated gene cloned in the pMK2 vector or carrying the empty vector (EV) in the ***n****COX2^W56R^* background at 30 °C. Photographs were taken on the seventh day of growth. Only lactate-dependent growth is shown, but no improvement was observed on ethanol/glycerol.

Figure S2. **Overexpression of the *TYE7*, *RAS2*, and *COX12* genes enhance the respiratory growth of a strain expressing the *COX2^W56R^* gene from a centromeric plasmid both at 30˚C and 37˚C.** Serial dilution series at 30˚C (upper panels) and 37˚C (lower panels) showing the fermentative (glucose) and respiratory phenotype (lactate) of a wild-type strain (wt), the *∆cox2* + ***cen****COX2^W56R^* strain (***cen****COX2^W56R^*), and the three strains overexpressing (↑) the indicated gene or carrying the empty vector (EV) in the ***cen****COX2^W56R^* background photographs were taken on day five.

Figure S3. **Overexpression of the *TYE7*, *RAS2*, and *COX12* genes promotes the growth of a strain expressing the *COX2^W56R^* gene from the nucleus. A)** Serial dilution series showing the fermentative (glucose) and respiratory (lactate) growth phenotypes of a wild-type strain (wt), the *∆cox2* + ***e****COX2^W56R^* strain (***e****COX2^W56R^*) expressing the allotopic construct from a multicopy vector and the three strains overexpressing (↑) the indicated gene or carrying the empty vector (EV) in the ***e****COX2^W56R^* background at 30 °C. Photographs were taken on the fourth (*upper panels*) and on the seventh day of growth (*lower panels*). **B)** Antibodies against Cox2 and Zwf1 were used to immunodetect the corresponding proteins in total cellular extracts of the indicated yeast strains: wild type (wt); the *∆cox2* + ***e****COX2^W56R^* strain (***e***), the three strains overexpressing (↑) the indicated gene on a ***e****COX2^W56R^* background, and the control strain transformed with an empty vector (***e*** + EV). The precursor and mature proteins Cox2 or Cox2^W56R^ (28 kDa) are indicated. The anti-Zw1 antibody immunoreacts against a 57 kDa band which is used as a loading control. Quantification of technical replicates of immunoblots are represented by bar plots (mean ± SD, n = 3). Black and grey dotted lines indicate the mean and SD abundance of the wild-type protein. *Δcox2* data is not displayed.
