# Supplementary material for: A high copy suppressor screen identifies factors enhancing the allotopic production of subunit II of cytochrome c oxidase
Source: G3 (Bethesda). 2024 Dec 13;15(3):jkae295. doi: 10.1093/g3journal/jkae295 (PMC11917479; doi:10.1093/g3journal/jkae295)
Supplement: jkae295_Supplementary_Data [file jkae295_supplementary_data.zip › Table_S1_G3-2024-405571.docx]

| **Strain** | **Alias** | **Genotype** | **Reference or source** |
| --- | --- | --- | --- |
| NB40-36A | wt | *MATα ;*  *lys2* ; *leu2-3,112 ; arg8::hisG* ; *ura3-52* [*rho^+^*] | Pérez-Martinez et al., 2003 |
| EHW154 |  | *MATa ; arg8::hisG ; his3-ΔHindIII ; leu2-3,112 ; lys2 ; ura3-52 ;* [*rho^+^*] *cox2(1,89-91)::arg8m* | Williams and Fox, 2003 |
| DRG102 EHW154 | ***n***COX2^W56R^ | *MATa ; arg8::hisG ; his3-ΔHindIII ; leu2-3,112 ; lys2 ; ura3-52 ; ura3-2::hphNT1-5PGK-COX2^W56R^-3PGK;* [*rho^+^*] *cox2(1,89-91)::arg8m* | Rubalcava-Gracia et al. 2019 |
| DRG103 EHW154 | ***n***COX2 | *MATa ; arg8::hisG ; his3-ΔHindIII ; leu2-3,112 ; lys2 ; ura3-52 ; ura3-2::hphNT1-5PGK-COX2-3PGK;* [*rho^+^*] *cox2(1,89-91)::arg8m* | Rubalcava-Gracia et al. 2019 |
| DRG104 EHW154 | ***n^uv^***COX2^W56R^ | *MATa ; arg8::hisG ; his3-ΔHindIII ; leu2-3,112 ; lys2 ; ura3-52 ; ura3-2::hphNT1-5PGK-COX2^W56R^-3PGK;* [*rho^+^*] *cox2(1,89-91)::arg8m* | This study |

**Table S1**

**STRAINS USED IN THIS STUDY**

Notes:

The yeast strains EHW154 and NB40-36A used in this work are congenic, since both originated in the same sporulation event of two strains D273-10b (ATCC 25627) (Bonnefoy and Fox, 2000).

*COX2^WT^* and *COX2^W56R^* correspond to the engineered strains described earlier (Supekova et al., 2010). 5PGK (821 bp) and 3PGK (260 bp) are the promotor and terminator sequences of PGK used to express genes in pFL61 (Minet et al., 1992).
