# Supplementary material for: A high copy suppressor screen identifies factors enhancing the allotopic production of subunit II of cytochrome c oxidase
Source: G3 (Bethesda). 2024 Dec 13;15(3):jkae295. doi: 10.1093/g3journal/jkae295 (PMC11917479; doi:10.1093/g3journal/jkae295)
Supplement: jkae295_Supplementary_Data [file jkae295_supplementary_data.zip › Table_S2_G3-2024-405571.docx]

**Table S2**

**Sequence, ID, product size and efficiency of the oligonucleotides used in qPCR amplification and quantitative determination of transcripts.**

| **GENE NAME** | **SGD ID** | **CODE NAME** | **SEQUENCE (5´→3´)** | **PRODUCT SIZE**  **(bp)** | **EFFICIENCY (%)** |
| --- | --- | --- | --- | --- | --- |
| *TYE7* | S000005871 | qTYE7_FW | CATCCAGCTCCTCGATAGGC | 131 | 95.8 |
| *TYE7* | S000005871 | qTYE7_RV | TGCTAACCTCGGGAGAATGC |  |  |
| *RAS2* | S000005042 | qRAS2_FW | GCACAACTGTCGTGAATGC | 76 | 108.4 |
| *RAS2* | S000005042 | qRAS2_RV | CCATTTGTCGCCTGGTTG |  |  |
| *COX1* | S000007260 | qCOX1_FW | GTGGTATGGCAGGAACAGC | 160 | 99.1 |
| *COX1* | S000007260 | qCOX1_RV | CCTCCAATTAAAGCAGGC |  |  |
| *COX3* | S000007283 | qCOX3_FW | GCATGTTGACCACCCGTAG | 123 | 109.9 |
| *COX3* | S000007283 | qCOX3_RV | CCTGCGATTAAGGCATGATGAC |  |  |
| *COX12* | S000004028 | qCOX12_FW | TGAAGGGCGAAGATTTTGCTC | 75 | 97.7 |
| *COX12* | S000004028 | qCOX12_RV | CGATCCAGTCTAGGGGACATAAG |  |  |
| *ZWF1* | S000005185 | ZWF1_FW | ACATCGAAAACGAGCGTTGG | 74 | 106.0 |
| *ZWF1* | S000005185 | ZWF1_RV | CACCTTGGACTCATTCAAAGCC |  |  |
| *TDH3* | S000003424 | TDH3-F | CAAGGAAACCACCTACGA | 99 | 103.9 |
| *TDH3* | S000003424 | TDH3-R | CGAAGATGGAAGAGTGAGAG |  |  |
| *CDC19* | S000000036 | CDC19-F | CAGAGGTGACTTGGGTATTG | 105 | 93.5 |
| *CDC19* | S000000036 | CDC19-R | GGTTGGTCTTGGGTTGTAAG |  |  |

Efficiencies were calculated from the slopes (m) of the standard curves in qPCR:

$$E={(10}^{-m}-1)x100$$
