# Supplementary figures and images for: A high copy suppressor screen identifies factors enhancing the allotopic production of subunit II of cytochrome c oxidase
Source: G3 (Bethesda). 2024 Dec 13;15(3):jkae295. doi: 10.1093/g3journal/jkae295 (PMC11917479; doi:10.1093/g3journal/jkae295)

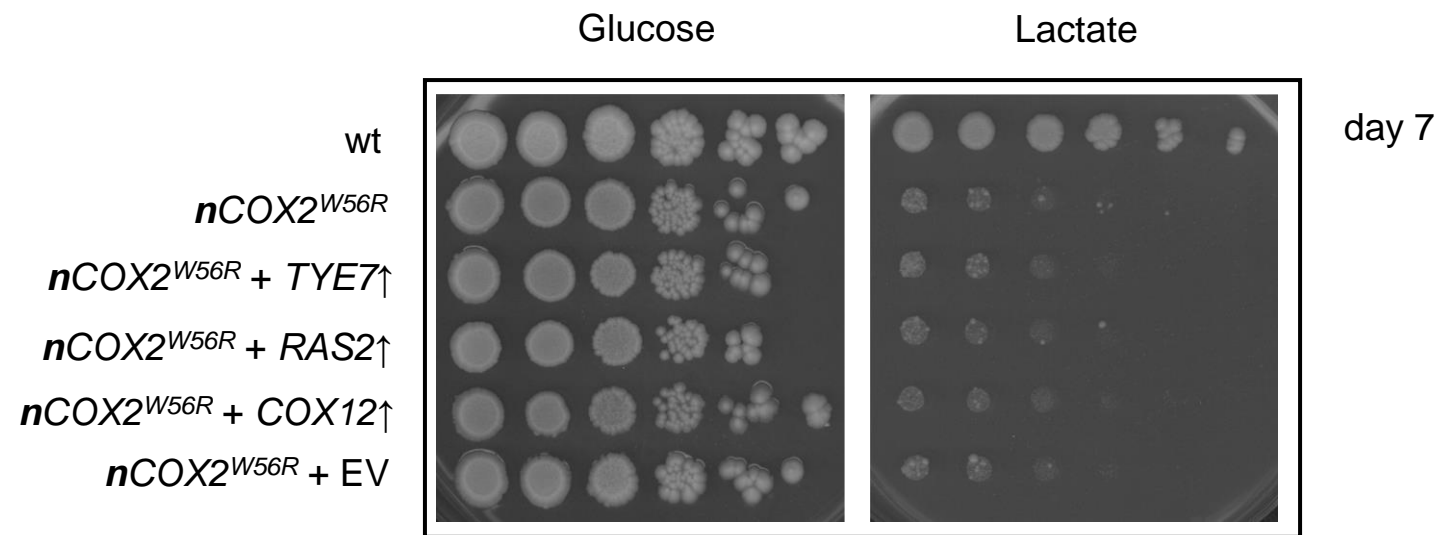

Figure S1

Supplement: jkae295_Supplementary_Data [file jkae295_supplementary_data.zip › Fig_S1_G3-2024-405571.pdf]

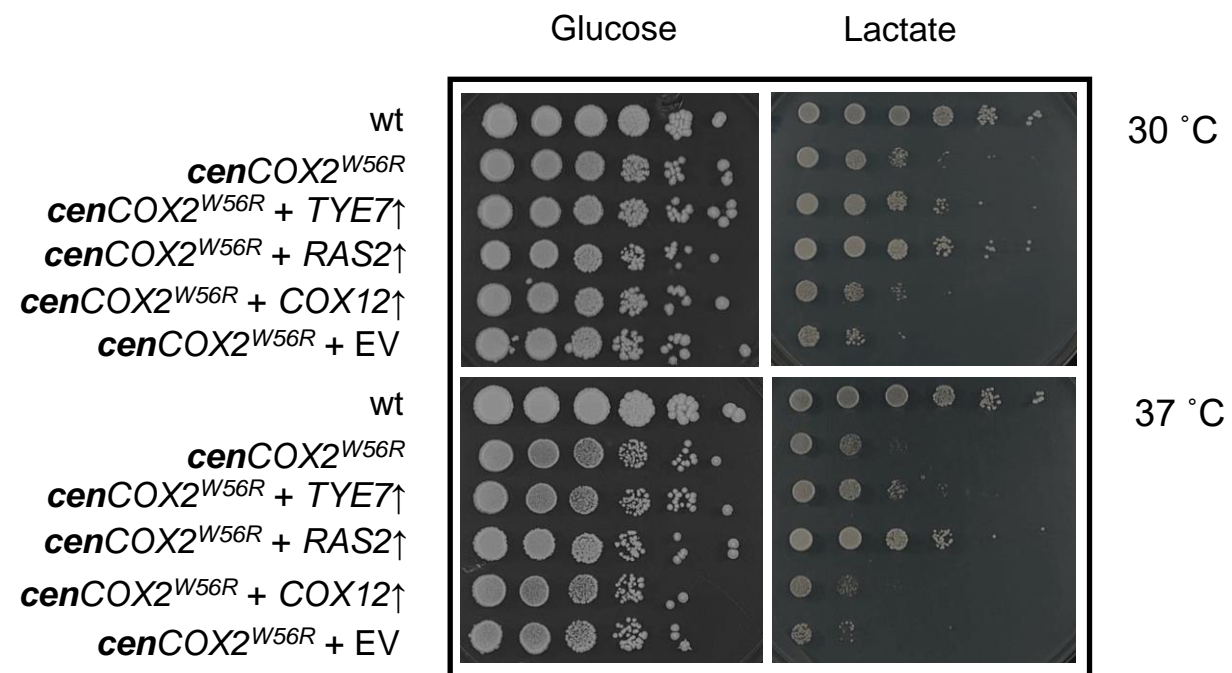

Figure S2

Supplement: jkae295_Supplementary_Data [file jkae295_supplementary_data.zip › Fig_S2_G3-2024-405571.pdf]

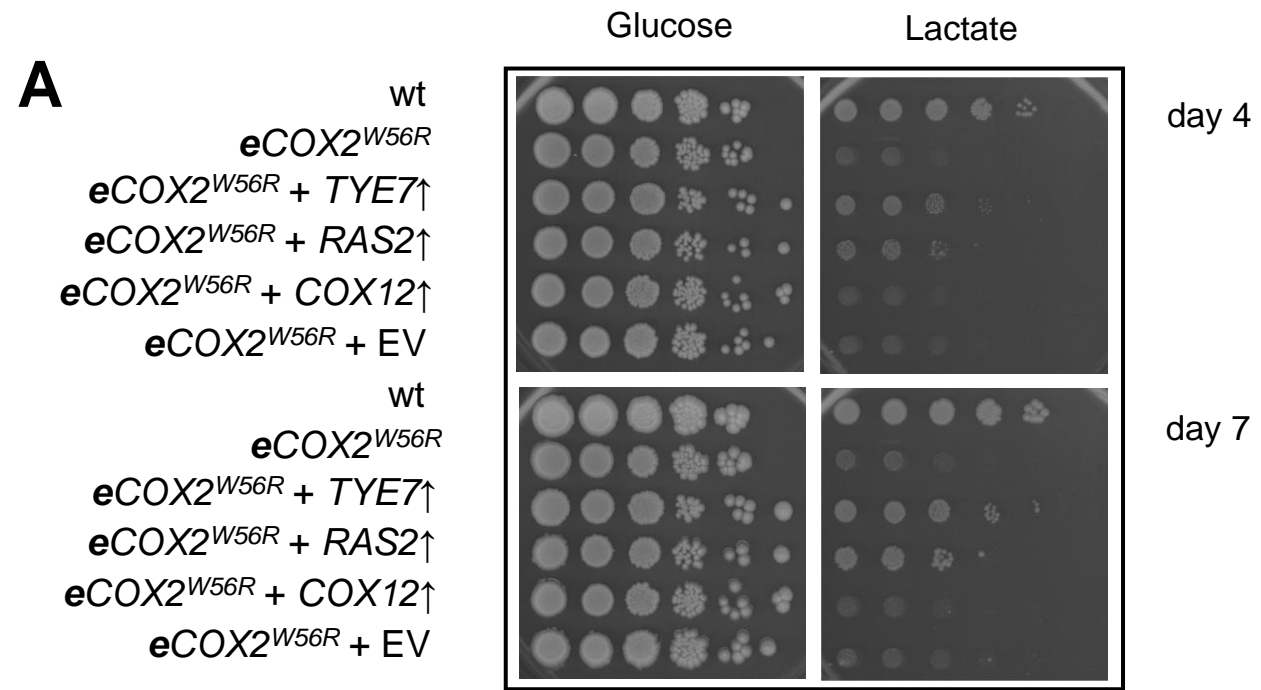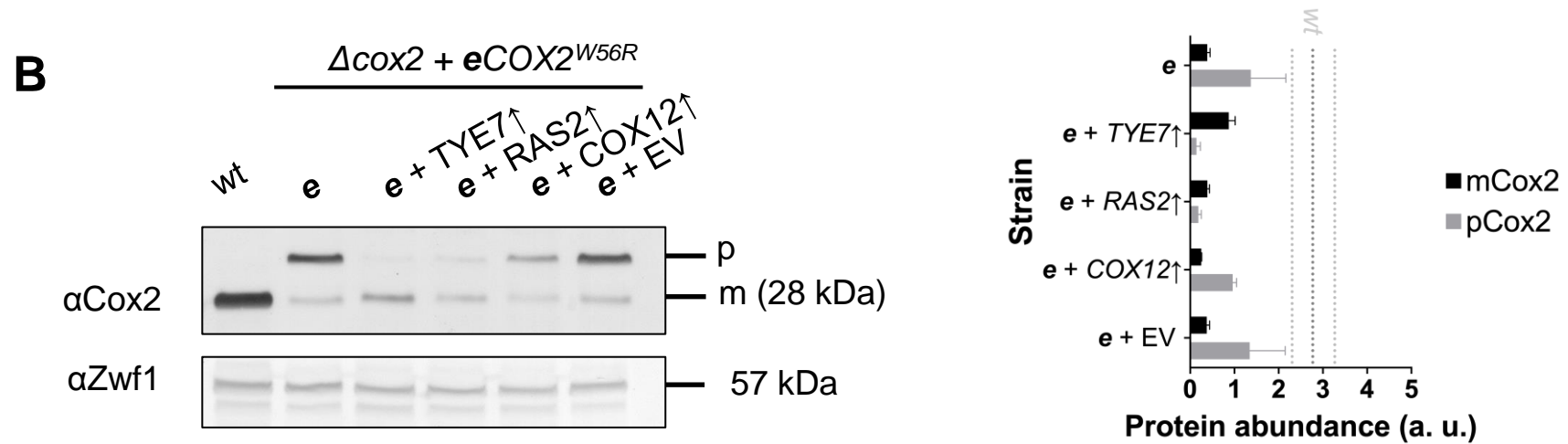

Figure S3

Supplement: jkae295_Supplementary_Data [file jkae295_supplementary_data.zip › Fig_S3_G3-2024-405571.pdf]
